# Risk Factors of Peritoneal Recurrence and Outcome of Resected Peritoneal Recurrence After Liver Resection in Hepatocellular Carcinoma: Review of 1222 Cases of Hepatectomy in a Tertiary Institution

**DOI:** 10.1245/s10434-012-2260-3

**Published:** 2012-03-09

**Authors:** Alfred Wei Chieh Kow, Choon Hyuck David Kwon, Sanghyun Song, Milljae Shin, Jong Man Kim, Jae-Won Joh

**Affiliations:** 1 Department of HPB Surgery and Liver Transplantation, University Surgical Cluster, National University Health System, Singapore, Singapore; 2 Department of Surgery, Samsung Medical Center, Sungkyunkwan University School of Medicine, Seoul, South Korea

## Abstract

**Background:**

Reports on the risk factors of peritoneal recurrence (PR) after liver resection for hepatocellular carcinoma are lacking. We examined the risk factors of PR after hepatectomy and the outcome of resected PR at our institution.

**Methods:**

We retrospectively reviewed the data from 1,222 patients who underwent hepatectomies for hepatocellular carcinoma in Samsung Medical Center from January 2006 to August 2010. We identified patients with PR and studied the risk factors and outcomes of resected PR.

**Results:**

The rate of PR was 3.0% (*n* = 36). The mean ± SD age of patients was 54.0 ± 10.2 years. Among those with PR, 23 patients (63.9%) had unresectable disease and 13 patients (36.1%) had resectable disease. Multivariate analysis found that tumor size >50 mm, presence of microvascular invasion, bile duct invasion, and positive margins were significant risk factors of PR after liver resection. The median overall survival (OS) for resectable PR was 33.0 (28.0–61.6) months compared to 14.0 (6.8–21.2) months for unresectable PR (*P* = 0.009). Cox regression analysis demonstrated that resected PR [hazard ratio (HR) 0.042, *P* = 0.001] and interval between hepatectomy and PR (>6months) (HR 0.195, *P* = 0.016) were positive prognostic factors for OS, while alfa-fetoprotein >200 ng/dl at detection of PR (HR 11.321, *P* = 0.015) and serosal involvement of primary hepatocellular carcinoma (HR 25.616, *P* = 0.007) were negative prognostic factors for OS.

**Conclusions:**

We found that tumor size >50 mm, presence of microvascular invasion, bile duct invasion, and positive resection margins were significant risk factors of PR after liver resection. Selected patients with resected PR had significantly better OS.

Hepatocellular carcinoma (HCC) is a highly prevalent malignancy, affecting approximately over half a million people worldwide.^1^ In Korea, HCC is the third leading malignancy after stomach and lung cancers. According to 2005 statistics, the age-adjusted incidence of primary HCC is 44.9 per 100,000 men.^2^,^3^ Chronic hepatitis B is endemic in Korea, representing the most important risk factor, constituting approximately 70% of all cases of HCC.^4^,^5^ Although liver transplantation for HCC has altered the natural history of this disease, liver resection remains one of the main treatment modalities that provides good outcome.^6^–^10^

Peritoneal implantation in HCC is uncommon, with reported incidences ranging 2–16%.^11^–^14^ Generally, peritoneal recurrence (PR) in HCC was considered to be an incurable terminal disease. Patients are rarely suitable for surgical resection of PR in HCC. Reports on the long-term outcome PR resection are lacking in the literature. A few anecdotal case reports have documented satisfactory outcome after surgical resection of PR in HCC.^15^–^22^ One of the largest series was a report from a Taiwanese center, reporting a median survival of 16 months in patients who underwent resection of peritoneal implantation.^23^ However, studies on the risk factors of PR and resected peritoneal lesions remain rare in the literature. There is a lack of recommendations in the management of PR in HCC after resection.

Selected patients with PR after liver resection underwent resection of the peritoneal lesions at our institution. Our primary aim was to investigate the risk factors influencing PR in HCC after liver resection. A secondary aim was to determine the predictive factors for resected PR after liver resection and its long-term outcome. From this information, we drew up guidelines for resection of PR in HCC after hepatectomy.

## Patients and Methods

We performed retrospective data collection of all patients who underwent liver resection for HCC at Samsung Medical Center, South Korea, from January 2006 to September 2010. After excluding patients who were lost to follow-up, 1,222 cases were suitable for analysis. During the process of data collection, we identified patients with PR documented by imaging studies and those who underwent resection of PR.

Demographic details (including the causative factor of HCC) and prehepatectomy tumor marker levels [alfa-fetoprotein (AFP) and protein induced by vitamin K absence or antagonist-II (PIVKA-II)] and other treatment modalities (e.g., radiofrequency ablation, transarterial chemoembolization) were collected from patients’ electronic medical records. Surveillance of patients with HCC after liver resection was performed at 3 month intervals for the first 2 years and then every 4–6 months thereafter. During the visit, tumor markers such as AFP and PIVKA-II were assayed. Elevated tumor markers prompted the suspicion of tumor recurrence, and further investigations were performed to ascertain the diagnosis. Options of the radiologic imaging included computed tomographic scan of the abdomen and pelvis (with computed tomography of the thorax), magnetic resonance imaging, and/or positron emission tomography scan.

The aim of the investigations was twofold. First, the investigations would help determine the locations of recurrences, number of lesions, and whether they were resectable. Second, the presence of extra-abdominal metastases that would preclude further curative resection would be ascertained. In the absence of other systemic metastasis or inoperable intrahepatic recurrences of HCC, if the peritoneal lesions were resectable and without compromising essential anatomic structures such as major vasculature, surgical resection would be offered to the patient.

After ascertaining that PRs were suitable for resection, the next step was to ensure that the selected patients were fit to undergo the operation. Careful preoperative assessment was carried out. At the operation, a midline incision was made to facilitate exploratory laparotomy. Careful adhesiolysis was performed to avoid enterotomy when adhesion was encountered. Locations and number of lesions were confirmed with the preoperative scans. Resection of the peritoneal lesions was carefully performed to ensure adequate surgical margin. En-bloc resection of the lesion, along with its surrounding structures, such as the small bowel or the cuff of diaphragm, was occasionally required. In situations where the malignant nature of the lesion was uncertain, excision for frozen section histology was performed intraoperatively. Careful hemostasis and selective placement of abdominal drains at surgical sites were performed.

Postoperative histologic assessment and reporting included maximum tumor diameter, Edmonson grade of HCC, microvascular invasion (MVI), portal vein/hepatic vein invasion, bile duct invasion, serosal involvement, intrahepatic metastasis, multicentric occurrence of HCC, and resection margin. tumor, node, metastasis system staging was performed in accordance with the American Joint Committee on Cancer (seventh edition) staging system.^24^ R0 resection status was defined as the presence of microscopic tumor >1 mm from the resection margin. There was no patient with R2 resection in this cohort.

Detailed information of patients who were identified to have PR was recorded. The interval from hepatectomy to PR was documented. For those who developed unresectable PR (commonly as a result of concurrent systemic metastasis to other sites such as lung and bone, or unresectable intrahepatic recurrence), radiologic information on the number, size, and site of peritoneal lesions was measured from the scan. For patients who underwent resection of PR, the operative and histologic data (including number and size of lesions, as well as the location of lesions) were clearly documented. Preoperative imaging findings on the number, size, and site of peritoneal lesions were also measured from the scan to allow comparison with unresectable PRs. Information on subsequent recurrence of peritoneal lesions and other sites of recurrence in this group of patients was also recorded. Overall survival (OS) was analyzed as the time from the date of hepatectomy until the date of death (i.e., death from all causes, including dead of disease and dead of other causes), whichever occurred first. Patients lost to follow-up or for whom the event of interest had yet been observed were censored at the date last known to be alive.

A total of 1,222 patients who underwent liver resection for HCC during the study duration were accrued. We studied the different parameters between two groups of patients with and without PR after liver resection. We classified the patients into those without PR (NPR) and those with PR. Further analysis of resected and unresectable PR was performed.

Data were analyzed by SPSS software, version 17.0 (SPSS, Chicago, IL). Statistical significance was set at a *P* value of <0.05. The χ^2^ test was used in cross-tabulation of nominal data. Student’s *t* test was performed for parametric continuous variables, and the Mann-Whitney *U* test was used for nonparametric continuous variables. Logistic and linear regression analyses were used to investigate the predictors of PR. Kaplan-Meier analysis was used to depict the OS and difference in survival between study groups.

## Results

### Distribution of Patients and Pattern of Distribution of PR

The mean ± SD age of this cohort was 54.0 ± 10.2 years. There were 1,186 patients (97.0%) in the NPR group and 36 patients (3.0%) in the PR group. Within the PR group, 23 patients (63.9%) had unresectable PR, and another 13 patients (36.1%) underwent resection of PR.

The median time from hepatectomy to PR was 6.5 (1.0–102.0) months. The most common site of PR was at the omentum (*n* = 17, 47.2%). One-third of patients had a single lesion (*n* = 13, 36.1%). The median number of lesions was 2.0 (1.0–7.0) (Table 1).

**Table 1:** Pattern of distribution of PR in the study cohort (*n* = 36)

Distribution	Value
Location of PR	
Omentum	17 (47.2%)
Posterior peritoneum^a^	11 (30.6%)
Anterior peritoneum^b^/abdominal wall	4 (11.1%)
Combination	4 (11.1%)
Posterior peritoneum with omental nodules	3 (8.3%)
Posterior peritoneum with chest wall	1 (2.8%)
No. of lesions	
Single peritoneal lesion	13 (36.1%)
Multiple peritoneal lesions	23 (63.9%)
Median no. of lesions	2.0 (1.0–7.0)
Size of lesion or lesions (mm)	
Median total size	48.5 (10.0–255.0)
Median largest size	25.5 (4.0–140.0)

^a^Posterior peritoneum included patients with PR located on diaphragmatic surface, posterior peritoneal lining near the right renal bed and posterior abdominal wall next to the chest wall

^b^Anterior peritoneum included anterior abdominal wall and 1 patient with tumor seeding on the serosa of sigmoid colon

### Analysis of Clinicopathologic Factors Influencing PR

The demographic data in the NPR and PR groups were not statistically significant. Nine patients (25%) in the PR group received other treatments before hepatectomy, compared to 189 patients (15.9%) in the NPR group (*P* = 0.426). There were significantly more patients in the PR group who had ruptured HCC (*n* = 2, 5.6%) before liver resection compared to the NPR group (*n* = 12, 1.0%) (*P* = 0.012). Slightly more than half (*n* = 624, 52.6%) of the liver resections in the NPR group were anatomic resections, compared to 69.4% (*n* = 25) in the PR group (*P* = 0.046) (Table 2).

**Table 2:** Univariate analysis of risk factors of PR (*n* = 1222)

Parameter	All	NPR group	PR group	*P*
	(*n* = 1222)	(*n* = 1186)	(*n* = 36)	
Age (year), mean ± SD	54.0 ± 10.2	54.0 ± 10.2	51.0 ± 10.3	0.545
Gender				
Male	977 (80.0%)	946 (79.7%)	31 (86.1%)	0.349
Female	245 (20.0%)	240 (20.2%)	5 (13.9%)	
Cause of HCC				
Chronic hepatitis B	940 (78.2%)	913 (78.4%)	26 (72.2%)	0.883
Chronic hepatitis C	62 (5.2%)	60 (5.1%)	2 (5.6%)	
Other (alcoholic liver disease, etc.)	200 (21.5%)	192 (16.5%)	8 (22.2%)	
History of ruptured HCC	14 (1.2%)	12 (1.0%)	2 (5.6%)	0.012*
Treatment before hepatectomy	198 (16.2%)	189 (15.9%)	9 (25.0%)	0.426
TACE	165 (83.3%)	158 (83.6%)	7 (77.8%)	0.879
RFA	18 (9.1%)	17 (9.0%)	1 (11.1%)	
Both RFA and TACE	15 (7.6%)	14 (7.4%)	1 (11.1%)	
Method of surgical access				
Open	1143 (93.8%)	1110 (93.8%)	33 (91.7%)	0.138
Laparoscopic	70 (5.7%)	68 (5.7%)	2 (5.6%)	
Laparoscopic converted to open	6 (0.5%)	5 (0.4%)	1 (2.8%)	
Types of hepatectomy				
Nonanatomic resection	573 (46.9%)	562 (47.4%)	11 (30.6%)	0.046*
Anatomic resection	649 (53.1%)	624 (52.6%)	25 (69.4%)	
Right hemihepatectomy	251 (20.5%)	238 (20.1%)	13 (36.1%)	0.051
Left hemihepatectomy	3 (0.2%)	2 (0.2%)	1 (2.8%)	
Posterior sectionectomy	125 (10.2%)	121 (10.2%)	4 (11.1%)	
Anterior sectionectomy	21 (1.7%)	20 (1.7%)	1 (2.8%)	
Extended right hepatectomy	30 (2.5%)	29 (2.4%)	1 (2.8%)	
Extended left hepatectomy	20 (1.6%)	20 (1.7%)	0 (0.0%)	
Left lateral sectionectomy	137 (11.2%)	134 (11.3%)	3 (8.3%)	
Anatomic monosegmentectomy	13 (1.1%)	13 (1.1%)	0 (0.0%)	
Central hepatectomy	49 (4.0%)	47 (4.0%)	2 (5.6%)	
Bisegmentectomy	67 (5.5%)	65 (5.5%)	2 (5.6%)	
Subsegmentectomy	506 (41.4%)	497 (41.9%)	9 (25.0%)	
Preoperative blood parameters				
Prehepatectomy AFP level, mean ± SD	5759.3 ± 64351.8	5391.8 ± 64691.7	17785.8 ± 51292.2	0.186
AFP ≤ 400 ng/ml	922 (75.9%)	898 (76.2%)	24 (66.7%0	0.189
AFP > 400 ng/ml	293 (24.1%)	281 (23.8%)	12 (33.3%)	
Prehepatectomy PIVKA-II level, mean ± SD	209.3 ± 306.2	209.8 ± 307.2	192.5 ± 267.8	0.888
Size of HCC at hepatectomy, mm, median (range)	35.0 (2.0–210.0)	35.0 (2.0–210.0)	45.5 (13.0–170.0)	0.001*
Stratification of size of HCC				
Tumor ≤ 50 mm	892 (73.0%)	871 (73.4%)	21 (58.3%)	0.002*
Tumor > 50 mm	330 (27.0%)	315 (26.6%)	15 (41.7%)	
Edmonson grade				
I and II	1095 (91.6%)	1064 (91.5%)	31 (93.9%)	0.617
III and IV	101 (8.4%)	99 (8.5%)	2 (6.1%)	
T stage of HCC				
T1 and T2	963 (80.8%)	943 (81.3%)	20 (62.5%)	0.008*
T3 and T4	229 (19.2%)	217 (19.7%)	12 (37.5%)	
Combined HCC and IHCC	38 (3.1%)	35 (3.0%)	3 (8.3%)	0.067
Invasion/involvement				
Tumor capsule involvement	1024 (85.4%)	995 (85.4%)	29 (85.3%)	0.985
Microvascular invasion	645 (52.8%)	618 (52.1%)	27 (75.0%)	0.007*
Bile duct invasion	35 (2.9%)	31 (2.7%)	4 (11.8%)	0.002*
Portal vein invasion	90 (7.5%)	84 (7.2%)	6 (17.6%)	0.022*
Serosal involvement	15 (1.3%)	13 (1.1%)	2 (5.9%)	0.014*
Intrahepatic metastasis	165 (13.7%)	158 (13.5%)	7 (20.6%)	0.238
Multicentric occurrence	59 (4.9%)	58 (5.0%)	1 (2.9%)	0.590
Totally necrotic nodule	21 (1.7%)	18 (1.5%)	3 (8.3%)	0.002*
Resection margin, mm, median (range)	10.0 (0.0–80.0)	10.0 (0.0–80.0)	8.0 (0.0–45.0)	0.360
Resection				
R0	1183 (97.7%)	1152 (98.0%)	31 (88.6%)	<0.001*
R1	28 (2.3%)	24 (2.0%)	4 (11.4%)	

*TACE* transarterial chemoembolization, *RFA* radiofrequency ablation, *IHCC* intrahepatic cholangiocacinoma

* Statistically significant at *P* < 0.05

The median tumor size of HCC in the PR group was 45.5 (13.0–170.0) mm, significantly larger by 10.5 mm compared to the median tumor size of HCC in the NPR group (median 35.0 mm, range 2.0–210.0 mm, *P* = 0.001). The proportion of tumors >50 mm in size in the PR group was 41.7% (*n* = 15) compared to 26.6% (*n* = 315) in the NPR group (*P* = 0.002). There was a significantly larger proportion of T3 and T4 tumors in the PR group (*n* = 12, 37.5%) compared to the NPR group (*n* = 217, 18.7%) (*P* = 0.008). The rates of MVI (*P* = 0.007), bile duct invasion (*P* = 0.002), portal vein/hepatic vein invasion (*P* = 0.022), serosal involvement (*P* = 0.014), and totally necrotic nodule (*P* = 0.002) in the PR group were significantly higher compared to the NPR group. Furthermore, there was a significantly higher proportion of HCC specimens with positive resection margin in the PR group (*n* = 4, 11.4%) compared to the NPR group (*n* = 24, 2.0%) (*P* < 0.001).

### Multivariate Analysis for Risk Factors of PR

The above factors found to be statistically significant on univariate analysis were examined by multivariate analysis. By means of logistic regression, we found that tumor size >50 mm [odds ratio (OR) 1.368, *P* = 0.032], presence of MVI (OR 3.896, *P* = 0.023), bile duct invasion (OR 3.720, *P* = 0.025), and positive resection margins (OR 9.540, *P* = 0.042) were significant predictors of PR after liver resection for HCC in this cohort (Table 3).

**Table 3:** Multivariate analysis for risk factors of peritoneal recurrence of HCC after hepatectomy by logistic regression

Independent risk factor for PR	OR	95% CI	*P*
Ruptured HCC	3.800	0.668–21.929	0.132
Anatomic vs. nonanatomic resection	2.375	0.212–1.107	0.123
Tumor > 50 mm	1.368	1.023–3.112	0.032*
T stage of tumor (T1 and T2 vs. T3 and T4)	0.901	0.356–2.282	0.815
Positive resection margin	9.540	1.746–18.733	0.042*
Microvascular invasion	3.896	1.164–8.165	0.023*
Bile duct invasion	3.720	1.179–12.279	0.025*
Portal vein invasion	0.508	0.583–5.000	0.476
Serosal involvement	3.184	0.813–28.042	0.074
Totally necrotic nodule	0.596	0.316–24.602	0.440

*OR* odds ratio, *CI* confidence interval

* Significant at *P* < 0.05

### Information at Time of Detection of PR

Twenty-three patients (63.9%) had unresectable PR, while another 13 patients (36.1%) underwent resection of PR. The median interval between hepatectomy and PR in the resected group was 11.0 (range 1.0–102.0) months, 5.0 months longer than the unresectable group (median 6.0 months, range 1.0–43.0 months, *P* = 0.009). There were almost 20% more patients with a disease-free interval of >6 months in the resected PR group (61.5% in the resected group vs. 43.4% in unresectable group, *P* = 0.080) (Table 4).

**Table 4:** Information at the time of detection of PR

Clinicopathologic factors	Unresectable PR	Resectable PR	*P*
	(*n* = 23)	(*n* = 13)	
Interval between hepatectomy and PR, mo, median (range)	6.0 (1.0–43.0)	11.0 (1.0–102.0)	0.009*
Interval between hepatectomy and peritoneal			
Recurrence			
≤6 month	13 (56.5%)	5 (38.4%)	0.080
>6 month	10 (43.4%)	8 (61.5%)	
Tumor markers at PR			
AFP level, mean ± SD	16519.7 ± 45832.1	575.4 ± 1475.2	0.032*
AFT level			
≤200 ng/dl	13 (56.5)	10 (76.9)	0.221
>200 ng/dl	10 (43.5)	3 (23.1)	
PIVKA-II level			
Mean ± SD	407.0 ± 498.1	180.5 ± 348.8	0.034*
≤200 mAU/ml	11 (55.0)	10 (83.3)	0.102
>200 mAU/ml	9 (45.0)	2 (16.7)	
No. of peritoneal lesions, median (range)	2.0 (1.0–7.0)	1.0 (1.0–4.0)	0.044*
Single lesion	6 (26.1%)	7 (53.8%)	0.096
Multiple lesions	17 (73.9%)	6 (46.2%)	
Size of largest peritoneal lesion			
Median (range), mm	24.0 (4.0–60.0)	35.0 (10.0–140.0)	0.070
≤30 mm	16 (69.6)	7 (30.4)	0.069
>30 mm	5 (38.5)	8 (61.5)	
Total size of peritoneal lesion			
Median (range), mm	47.0 (20.0–255)	60.0 (10.0–140.0)	0.499
≤50 mm	14 (60.9)	9 (39.1)	0.196
>50 mm	5 (38.5)	8 (61.5)	
Location of PR			
Omentum	17 (73.9%)	0 (0.0%)	<0.001*
Posterior peritoneum	4 (17.4%)	7 (53.8%)	
Anterior peritoneal/ abdominal wall	2 (8.7%)	2 (15.4%)	
Combination	0 (0.0%)	4 (30.8%)	
Posterior peritoneum with omental nodules	–	3 (23.1%)	
Posterior peritoneum with chest wall	–	1 (7.7%)	
Omental seeding	17 (73.9%)	3 (23.1%)	<0.003*
Other	6 (26.1%)	10 (76.9%)	

* Statistically significant at *P* < 0.05

At the time of detection of PR, the median number of lesion in the unresectable group was 2.0 (range 1.0–7.0), compared to 1.0 (range 1.0–4.0) in the resected group (*P* = 0.044). Although we were unable to demonstrate statistical significance, the proportion of patients with solitary lesions in the resectable group (*n* = 7, 53.8%) was doubled that in the unresectable group (*n* = 6, 26.1%), with close to three-quarters of patients in the unresectable group having multiple peritoneal lesions at recurrence (*n* = 17, 73.9%) (*P* = 0.096). In terms of location, the most common site of peritoneal metastasis in patients with unresectable PR was at the omentum (73.9%); posterior peritoneal lining (including diaphragm) was the most common site in resected PR (53.8%) (*P* < 0.001).

### Long-Term Outcome (Survival Analysis) of PR

**Fig. 1:**
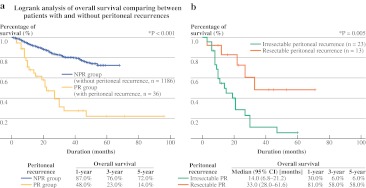
**a** OS of HCC patients comparing the NPR group and the PR group after hepatectomies at a tertiary institution. **b** OS of patients with resectable and unresectable PR after hepatectomies for HCC

The OS for patients in the NPR group was significantly better compared to that in the PR group (*P* < 0.001). The 1-, 3-, and 5-year OS for the NPR group was 87.0, 76.0, and 72.0%, respectively, compared to that in the PR group, which was 48.0, 23.0, and 14.0%, respectively (Fig. 1a).

**Fig. 2:**
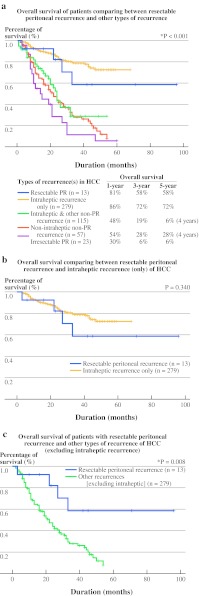
**a** Comparison of OS of resectable PR with other types of recurrence of HCC after liver resection. **b** Comparison of OS between resectable PR with intrahepatic recurrence only for HCC after curative resection. **c** Comparison of OS of patients with resectable PR with other types of recurrence (excluding intrahepatic recurrence)

However, the median OS for resected PR was significantly longer (33.0 months) compared to patients with unresectable PR (14.0 months) (*P* = 0.009) (Fig. 1b). The 1-, 3-, and 5-year OS of patients with resected PR was 71.0, 48.0, and 48.0%, compared to 30.0, 6.0, and 6.0%, respectively, for patients with unresectable PR (*P* < 0.001). Figure 2a shows the OS for various groups of recurrences in comparison with resected and unresectable PR. Resected PR was found to have comparable OS compared with patients with only intrahepatic HCC recurrence (*P* = 0.340) (Fig. 2b) but was significantly better than patients with other types of nonintrahepatic HCC recurrence (*P* = 0.008) (Fig. 2c).

### Prognostic Factors of OS for Patients with PR

By univariate analysis, we found that resected PR (*P* = 0.005), prehepatectomy AFP > 400 ng/dl (*P* = 0.008), HCC size > 50 mm (*P* = 0.023), higher Edmonson grade (*P* = 0.033), serosal involvement (*P* = 0.002), interval between hepatectomy and PR > 6 months (*P* = 0.001), and AFP at detection of PR > 200 ng/dl (*P* = 0.008) were significant factors. The use of sorafenib was not found to have any survival benefit (*P* = 0.092) (Table 5).

**Table 5:** Univariate and multivariate analyses of overall survival in patients with PR

Factor	Univariate analysis			Multivariate analysis		
	HR	95% CI	*P*	HR	95% CI	*P*
PR resectable	0.207	0.069–0.621	0.005*	0.042	0.007–0.262	0.001*
Prehepatectomy AFP > 400 ng/dl	3.232	1.353–7.721	0.008*	0.458	0.067–3.120	0.425
Prehepatic PIVKA-II > 200 mAU/ml	2.124	0.805–5.604	0.128			
HCC size > 50 mm	2.645	1.142–6.126	0.023*	0.819	0.150–4.482	0.818
Edmonson grade III and IV	5.759	1.151–28.808	0.033*	0.156	0.023–1.041	0.055
Stratified T stage of HCC (III and IV)	1.882	0.795–4.456	0.150			
Microvascular invasion	2.157	0.721–6.452	0.169			
Portal vein invasion	2.344	0.845–6.500	0.102			
Bile duct invasion	1.603	0.533–4.826	0.401			
Serosal involvement	22.271	3.054–162.407	0.002*	25.616	2.436–269.313	0.007*
Positive resection margin	3.870	0.790–18.953	0.095			
Interval between hepatectomy and PR > 6 mo	0.181	0.064–0.514	0.001*	0.195	0.041–0.922	0.039*
AFP at detection of PR > 200 ng/dl	1.884	0.927–3.829	0.008*	11.321	1.766–72.581	0.010*
PIVKA-II at detection of PR > 200 mAU/ml	0.852	0.402–1.808	0.677			
Single vs. multiple peritoneal lesions	1.996	0.815–4.890	0.131			
Omental seeding vs. other locations	0.450	0.184–1.099	0.080			
Size of largest peritoneal lesion > 30 mm	1.363	0.594–3.125	0.465			
Total size of peritoneal lesions > 50 mm	1.096	0.482–2.495	0.827			

*CI* confidence interval

* Statistically significant at *P* < 0.05

By Cox regression analysis, we found that resected PR [hazard ratio (HR) 0.042, *P* = 0.001] and an interval between hepatectomy and PR of >6 months (HR 0.181, *P* = 0.001) were positive predictors of OS in patients with PR. On the other hand, serosal involvement at primary HCC (HR 25.616, *P* = 0.007) and AFP at detection of PR (HR 11.321, *P* = 0.010) had a significant negative influence on the survival of patients with PR.

### Information on Resected PR

Eight patients (61.5%) with resected PR were still alive at the study’s completion. Patients with fewer lesions and long intervals between hepatectomy and PR had a much better outcomes compared to those with numerous peritoneal lesions. Five patients (38.4%) experienced recurrent peritoneal lesions, but only one underwent further resection. Another patient had recurrence at the chest wall and underwent resection. Three patients survived more than 5 years. All three of them also had a long interval between hepatectomy and detection of PR (48, 52, and 102 months, respectively) (Table 6). One of them has since died.

**Table 6:** Information on resected PR

Patient no.	Details of resected PR	No. of lesions	Size of largest lesion (mm)	Total size of lesions (mm)	Interval between hepatectomy and PR (month)	Overall survival (month)	Survival status	Further recurrent peritoneal lesions
1	Peritoneal mass, abdominal wall nodule and subxiphoid mass	4	40	125	9	10	Alive	No
2	Peritoneal mass and omentum	4	50	86	1	3	Dead	Yes, resected chest wall lesion
3	Omentum, splenic hilum at lesser curve	3	50	130	19	33	Dead	Yes, not resectable
4	Lesser sac, caudate lobe and	3	35	70	2	6	Alive	Yes, resected peritoneal lesion
5	Right upper quadrant mass and right chest wall	2	50	100	18	45	Alive	Yes, not resectable
6	Omentum and diaphragmatic nodule	2	35	58	48	102	Alive	No
7	Subdiaphragmatic mass with en bloc resection of diaphragm	1	23	23	102	115	Dead	No
8	Abdominal wall mass	1	15	15	52	71	Alive	No
9	Right-sided abdominal wall mass	1	10	10	19	42	Alive	No
10	Peritoneal nodule	1	12	12	6	23	Alive	No
11	Abdominal wall at rectus abdominis	1	21	21	6	22	Dead	No
12	Anterior aspect of duodenum and peritoneum on head of pancreas	1	60	60	6	27	Dead	Yes, not resectable
13	Metastasis to SB and sigmoid colon	1	140	140	11	16	Alive	Yes, not resectable

## Discussion

After resection, tumor recurrence rate is exceedingly high in HCC, reaching about 70% at 5 years.^7^,^25^–^27^ Most of the recurrences occur within the liver. Common sites of systemic metastasis of HCC after resection are lungs, bones, and brain. Other systemic sites such as adrenal glands, spleen, pancreas, and ovaries are usually not detected until autopsy.^28^–^30^ PR in HCC is uncommon. The reported incidence ranged 2–16%.^11^–^14^,^31^ In our series, the PR rate was 3.0%. The rate of unresectable PR was 1.9%, while another 1.1% underwent resection of PR after liver resection.

Because PRs after hepatectomy for HCC are rare, many previous reports related to PRs were included in studies focusing on extrahepatic recurrences. In comparison, our study solely examined the risk factors of PR in the cohort of patients. Cha et al. from Memorial Sloan-Kettering Cancer Center reported that tumor size of >5 cm and presence of vascular invasion were significant predictors of extrahepatic recurrences in HCC after resection. These two factors were similarly found to be significant predictors for PR in our cohort. Furthermore, a positive resection margins was not found to be a significant factor as a result of its low frequency in their study.^32^ In contrast, we found that positive resection margin was a significant predictor of PR, whereas presence or portal vein/hepatic vein invasion was not. Furthermore, the presence of bile duct invasion was found to increase the risk of PR in our study. In terms of the pattern of distribution of PR, we noted that the omentum was the most common site (47.2%), followed by the posterior peritoneal lining (inclusive of parietal peritoneal surface of diaphragm). This was consistent with the pattern observed in the study by Yeh et al., in which omental nodules constituted 50% of all cases.^23^ However, it is important to emphasize that our series included both resectable and unresectable PR, whereas Yeh et al. studied only resected PR patients.

Ruptured tumor has commonly been postulated to increase the risk of PR in HCC. Most of the reports on peritoneal metastasis after ruptured HCC were case reports. Many of the reports note that peritoneal metastases occurred 8 months after tumor rupture, although some occurred as early as 3–4 months after ruptured HCC.^33^,^34^ In our study, there were only two patients in the PR group with a history of ruptured HCC. One of them developed four isolated peritoneal lesions 1 month after hepatectomy and underwent resection of those lesions (because no other distant metastasis was noted). Another patient developed seven disseminated peritoneal nodules with multiple intrahepatic lesions at 6 months after liver resection after ruptured HCC and was treated conservatively. This differs greatly from many reports suggesting that a single metastatic nodule is the most common presentation for PR after ruptured HCC.^33^,^34^ Shin et al. reported a patient who survived a long time with recurred HCC after three bouts of repeated hepatic resection for intrahepatic recurrences and one bout of right hemicolectomy for an omental recurrence.^35^ In the current study, ruptured HCC was a significant factor for PR by univariate analysis but did not show significance on multivariate analysis, probably as a result of the low incidence of ruptured HCC in our cohort (*n* = 14, 1.2%).

Tumor markers levels, such as prehepatectomy PIVKA-II, AFP, and PIVKA-II level at detection of PR, were all significantly higher in patients with unresectable PR. This could indicate that the higher tumor load and the disseminated nature of the disease could preclude them from undergoing potentially curative surgical resection of PR. Indeed, most of the patients with unresectable PR had concomitant intrahepatic tumor recurrence and/or other systemic metastases. We also found that patients with smaller HCC (≤50 mm) and lower T stage of tumor at primary hepatectomy were more likely to undergo resection of PR. In addition, they also had significantly fewer lesions (median 1.0, range 1.0–4.0) compared to those resectable disease (median 2.0, range 1.0–7.0). In this regard, Yeh and Chen reported that tumor capsule invasion and elevated AFP level >400 ng/ml were significant predictors of peritoneal implantation after hepatectomy.^13^

Reports on the long-term survival outcome of patients with peritoneal metastasis after hepatectomy are scarce in the literature, leading to a lack of recommendations in the management of PR in HCC. In general, when PR occurs in HCC, surgical resection with curative intent would not be considered. In one rare report of 20 year data by Lin et al., patients with metachronous peritoneal metastasis experienced a median survival of 12.5 months—significantly longer than those who did not undergo PR resection (2.1 months).^14^ In addition, Nakayama et al. reported that resection of peritoneal metastases arising from HCC is justified because patients experienced disease-free survival and OS of 11.8 to 81.6 months after salvage therapy.^12^ In our series, the median OS for resected PR was 33.0 months, which was significantly longer than patients with unresectable PR (median OS 15.0 months). The 5 year survival for resectable PR was 48.0%, compared to 6.0% in patients with unresectable PR.

Some patients with PR after resection of HCC may experience good long-term survival if the peritoneal metastasis was resected. In the current study, in addition to resection of the peritoneal lesions, longer interval between hepatectomy and detection of PR > 6 months was a positive prognostic factor for OS. On the other hand, presence of serosal involvement and high AFP levels of >200 ng/dl at detection of PR were significant negative predictors of survival in PR. Indeed, a long disease-free interval suggested that there was a subset of patients with better tumor biology, and thus the peritoneal metastasis took a much longer time to become clinically evident. This group of patients would certainly experience better long-term survival after peritoneal metastasectomy. There were a few long-term survivors in our series with disease-free intervals of more than 48 months and an OS of more than 5 years. Two were still alive at the time of last follow-up.

Although resection of HCC was associated with a high incidence of recurrence, aggressive therapy for the recurrence is a key strategy in prolonging survival. On the basis of our analysis, we strongly recommend that surgical resection of PR be offered as an option as part of a multimodality therapy in HCC after hepatectomy in selected patients. Suitable candidates include patients with small tumor size (HCC ≤ 50 mm), low T stage of tumor, prehepatectomy PIVKA-II level of <200 mg/dl, low AFP level at detection of PR (<200 ng/dl), and long interval between hepatectomy and PR (>6 months).

Finally, we acknowledge that the retrospective nature of this analysis is a limitation of the study. The study could potentially underestimate the real incidence of PR, which was the reason why we reviewed such a large series of patients and went through their post-HCC resection scans in such detail. Further prospective studies would result in a better understanding of this condition. However, we recognize that this would be a difficult task because of the low incidence of PR in HCC.

In conclusion, peritoneal metastases of HCC are rare. Risk factors that we identified as predictors of PR after hepatectomy for HCC included tumor size >50 mm, presence of MVI, bile duct invasion, and positive resection margins. Selected patients with small tumor size (HCC ≤ 50 mm), low T stage of tumor, prehepatectomy PIVKA-II level <200 mg/dl, low AFP level at detection of PR (<200 ng/dl), and long interval between hepatectomy and resected PR (>6 months) would be suitable candidates for resection of PR.
